# Anatomy of Bluetongue virus Serotype 8 Epizootic Wave, France, 2007–2008

**DOI:** 10.3201/eid1612.100412

**Published:** 2010-12

**Authors:** Benoit Durand, Gina Zanella, Fabienne Biteau-Coroller, Caroline Locatelli, Florence Baurier, Cécile Simon, Eric Le Dréan, José Delaval, Eric Prengère, Véronique Beauté, Hélène Guis

**Affiliations:** Author affiliations: Agence Française de Sécurité Sanitaire des Aliment, Maisons-Alfort, France (B. Durand, G. Zanella);; Centre International de Recherche Agronimique pour le Développement, Montpellier, France (F. Biteau-Coroller, H. Guis);; Laboratoire Départemental d’Analyses 08, Hagnicourt, France (C. Locatelli);; Laboratoire Départemental d’Analyses 18, Bourges, France (F. Baurier, C. Simon);; Laboratoire Départemental d’Analyses 35, Rennes, France (E. Le Dréan);; Laboratoire Départemental d’Analyses 36, Chateauroux, France (J. Delaval);; Laboratoire Départemental d’Analyses 37, Tours, France (J. Delaval, E. Prengère);; Laboratoire Départemental d’Analyses 49, Angers, France (V. Beauté)

**Keywords:** Viruses, bluetongue, vector-borne infections, epizootic, France, serology, cattle, sheep, research

## Abstract

The introduction of bluetongue virus serotype 8 into northern Europe at the end of summer 2006 initiated one of the most widespread epizootics of bluetongue infection ever to occur. In winter 2007–2008, a cross-sectional serologic study was conducted in France along a transect perpendicular to the epizootic wave. Cattle herd-level seroprevalence varied from 4% to 100%, and animal-level seroprevalence from <1% to 40%. Only a low proportion of seropositive herds reported clinical cases in 2007. Sheep flocks were less frequently affected than cattle herds. The local occurrence of clinical cases and environmental indicators linked to forests were seropositivity risk factors, whereas the local density of cows had a protective effect. Overall results suggest that amplification of virus circulation in affected herds played a limited role in the epizootic wave diffusion and that bluetongue virus serotype 8 circulation in natural ecosystems could have played a substantial role in this progression.

## Abstract 2

Environmental seropositivity risk factors indicate natural ecosystems may have affected spread of the disease.

Bluetongue is a vector-borne viral disease of wild and domestic ruminants caused by *Bluetongue virus* (BTV; family *Reoviridae*, genus *Reovirus*). Twenty-four serotypes of this virus are described, principally transmitted by several species of biting midges belonging to the genus *Culicoides* (Diptera: Ceratopogonidae). Until 1998, Europe was considered BTV free except for occasional incursions into the Iberian Peninsula, Cyprus, and Greek islands. In 1998, an unprecedented series of successful introductions of BTV serotypes occurred in countries in southern, western, and central Europe (*1*). Unexpectedly, in 2006, BTV serotype 8 (BTV-8) was introduced in Belgium, close to the borders with Germany and the Netherlands (*2**,**3*), and quickly spread in these 3 countries. By the end of 2009, BTV-8 had spread to most countries in western and central Europe, including the United Kingdom, Denmark, Norway, Sweden, Czech Republic, Hungary, Austria, Italy, Luxembourg, Spain, and France.

In France, the first clinical case was reported in late August 2006 near the border with Belgium. In July 2007, bluetongue reappeared there (*4*) and quickly progressed westward and southward, with the virus causing clinical cases in 10,500 herds in 2007 and in 26,500 herds in 2008. By the end of 2007, BTV-1 was introduced in southern France, resulting in a second epizootic wave that progressed northward during 2008; by late 2008, most of the French territory had been affected by BTV-1, BTV-8, or both serotypes (Video). A vaccination campaign launched in 2008 stopped the epizootic in 2009. During the winter of 2007–2008, the end of BTV transmission (during the vector inactivity period) offered the opportunity to study the epizootic wave. The objectives of this study were to describe the first of these 2 epizootic waves and to analyze the respective parts played by within- and between-herd dynamics in BTV-8 progression, the relationship between the progression of infection and that of clinical cases, and the environmental features that influenced the progression of BTV-8.

**Video:**
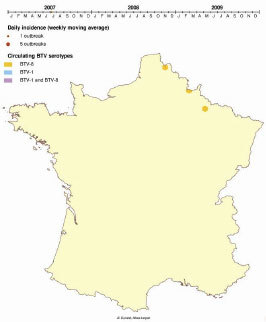
Movement of bluetongue virus in France, 2007–2009. A serotype 8 epizootic wave originated in northern France and progressed southward during 2007–2009; a serotype 1 epizootic wave began in southern France in 2007 and progressed northward during 2008–2009.

## Materials and Methods

Seroprevalence rates were estimated at the herd and animal levels in cattle and sheep along an east–west transect perpendicular to the epizootic wave. By comparing serologic results with clinical outbreaks, we estimated the proportion of silently infected herds or flocks (i.e., herds in which BTV-8 had circulated without any reported clinical cases) and variations in the outbreak along the transect. Main herd-level seropositivity risk factors were investigated (species, occurrence of clinical cases), as well as local seropositivity risk factors (animal density, land cover and landscape indices, occurrence of clinical cases).

### Study Area and Sampling Design

**Figure 1:**
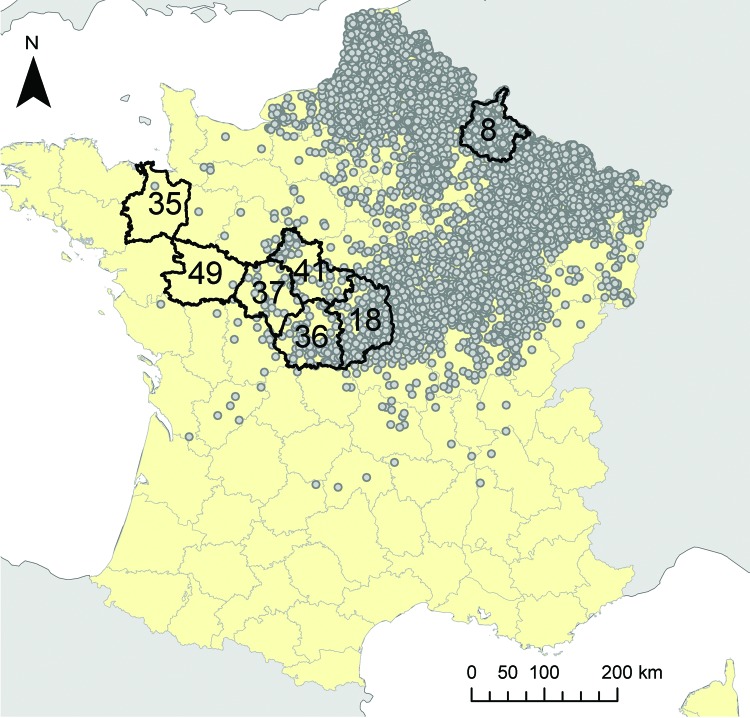
Locations included in a serologic study of the 2007 epizootic wave of bluetongue virus serotype 8 (BTV-8) among cattle herds in France. Black lines indicate the 7 departments included in the study: 6 departments aligned on an east–west transect (codes 18, 41, 36, 37, 49, and 35); and the first department to report BTV-8 infection in 2006 (code 08). Dots represent locations of BTV-8 outbreaks during 2007.

The study area comprised 7 departments in France (Figure 1); 6 departments situated on an east–west transect from the center of the country to Brittany (codes 18, 41, 36, 37, 49, and 35) and, as a reference, the department where the first outbreak in France was reported in late 2006 (code 08). Sample size was calculated for a herd-level design prevalence of 10% and a precision of ±10%. Within-herd design prevalence was set to 10% with a detection probability (>1 seropositive animal) of 95%. We sampled 50 herds per department, with 30 animals tested in each herd. This sampling protocol was applied to the beef cattle population in the 7 selected departments and to the sheep population in 4 of these departments selected because of the linear decrease (on a logarithmic scale) of the number of outbreaks reported in 2007 (department 08: 1,405 outbreaks, department 18: 104 outbreaks, department 36: 14 outbreaks, and department 35: 1 outbreak).

### Biological Samples and Laboratory Analyses

The study was conducted in close collaboration with local veterinary laboratories. The laboratories were in charge of randomly drawing samples of herds and animals from their banks of serum. The sampling base was the set of serum samples taken during winter 2007–2008 for brucellosis detection, which is mandatory in France for beef cattle >12 months of age and for small ruminants >6 months of age. The sampling protocol was satisfactorily implemented, except in department 36 where only few sheep serum samples were available (Table 1, Table 2). Overall, in the 7 departments, 9,888 serum samples were tested from 360 beef cattle herds, and 2,465 serum samples were tested from 157 sheep flocks (Table 1, Table 2). All the local veterinary laboratories that participated in the study were accredited for detection of BTV-8 antibodies. Serum was analyzed by using a commercial ELISA kit according to the recommendations of the manufacturer (ID-Vet, Montpellier, France). ELISA specificity is high in clinically suspected disease in sheep and cattle (*5**,**6*), the lower sensitivity in these animals being attributed to early stages of infection (*6*). No estimate is available for healthy cattle and sheep. We thus assumed that sensitivity and specificity were perfect; this may have induced a slight underestimate of seroprevalence rates.

**Table 1:** Herd-level anti–bluetongue virus serotype 8 seroprevalence rate and clinical expression of bluetongue disease in 7 departments, France, winter 2007–2008*

Species	Department code	No. positive†/no. tested	Herd-level seroprevalence (95% CI‡)	No. outbreaks§	Reporting rate¶ (95% CI)
Cattle	08	63/63	1.00 (0.94–1.00)	37	0.59 (0.46–0.71)
	18	49/49	1.00 (0.93–1.00)	4	0.08 (0.02–0.20)
	41	42/50	0.84 (0.71–0.93)	3	0.07 (0.01–0.19)
	36	29/48	0.60 (0.45–0.74)	1	0.03 (0.00–0.18)
	37	31/50	0.62 (0.47–0.75)	0	0.00 (0.00–0.11)
	49	1/50	0.02 (0.00–0.11)	0	0.00 (0.00–0.98)
	35	2/50	0.04 (0.00–0.14)	0	0.00 (0.00–0.84)
Sheep	08	42/44	0.95 (0.85–0.99)	23	0.55 (0.39–0.70)
	18	14/59	0.24 (0.14–0.37)	2	0.14 (0.02–0.43)
	36	0/4	0.00 (0.00–0.60)	NA	NA
	35	0/50	0.00 (0.00–0.07)	NA	NA

*CI, confidence interval; NA, no seropositive flock. †No. seropositive herds: *>*1positive result among tested serum samples. ‡Exact binomial 95% CI. §No. seropositive herds with reported confirmed clinical disease in 2007. ¶Proportion of seropositive herds with reported confirmed clinical cases in 2007.

**Table 2:** Animal-level anti–bluetongue virus serotype 8 seroprevalence rate and distribution of within-herd seroprevalence rates in seropositive herds or flocks of 7 departments, France, winter 2007–2008*

Species	Department code	No. positive/no. tested	Animal-level seroprevalence (95% CI†)	Median within-herd seroprevalence in seropositive herds (25%–75% quartiles)
Cattle	08	1,563/1,573	0.99 (0.99–1.00)	1.00 (1.00–1.00)
	18	642/1,530	0.42 (0.39–0.44)	0.40 (0.23–0.57)
	41	203/1,500	0.16 (0.14–0.17)	0.15 (0.07–0.27)
	36	103/1,470	0.07 (0.06–0.08)	0.10 (0.07–0.17)
	37	103/1,500	0.07 (0.06–0.08)	0.10 (0.05–0.13)
	49	1/1,410	0.001 (0.000–0.003)	0.03‡
	35	2/905	0.002 (0.000–0.007)	0.03§
Sheep	08	478/833	0.57 (0.54–0.61)	0.70 (0.43–0.87)
	18	19/874	0.02 (0.01–0.03)	0.08 (0.06–0.10)
	36	0/326	0.00 (0.00–0.01)	NA
	35	0/432	0.00 (0.00–0.01)	NA

*CI, confidence interval; NA, no seropositive flock. †Exact binomial 95% CI. ‡ A single seropositive herd. §Two seropositive herds or flocks with the same within-herd seroprevalence.

### Outbreak, Population, and Land Cover Data

The database of BTV-8 outbreaks reported in France in 2007 was screened to determine whether confirmed clinical disease cases had been reported in the herds included in the study. Confirmed clinical cases were defined as disease in animals showing BTV-8 clinical signs and for which BTV-8 had been isolated or a positive BTV-8–specific PCR result had been obtained. For each herd we defined a binary variable, an outbreak status of 1 if a clinical case had been reported in 2007 and 0 if otherwise.

In the 6 departments of the east–west transect (Figure 1), each tested herd was geo-referenced at the municipality level (the smallest French administrative subdivision). For each municipality, we obtained from the French national database the number of cows and the number of flocks of small ruminants. Municipality-specific land cover data were extracted from the 2006 version of the CORINE (Coordination de l’Information sur l’Environnement) Land Cover database (CLC), provided by the European Environment Agency (*7*), at a 1:100,000 working scale (resolution of 100 m). The 44 classes of CLC nomenclature aim at describing perennial structures of land occupation and are organized into a general-purpose 3-level hierarchy. For our study, the first level of this hierarchy was used, except for the second class of this first level (agricultural areas) for which the second level (more detailed) nomenclature was used. In addition, themes related to water were separated into 2 classes for inland versus marine wetlands and water bodies. These modifications resulted in a nomenclature with 8 classes (Table 3). For each municipality, we computed the proportion of the area covered by each class (8 variables) and, for each pair of classes, the edge density: the length of edges between the 2 classes divided by the municipality area (28 variables expressed in 100 m/ha).

**Table 3:** Land cover repartition in municipalities with at least 1 herd tested, in 6 French departments aligned on an east–west transect*

ID	Land cover classes	CLC classes	Department†					
18	41	36	37	49	35
C_1_	Artificial surfaces	111–142‡	0.02	0.02	0.01	0.03	0.04	0.06
C_2_	Arable land	211–213	0.33	0.51	0.24	0.46	0.45	0.33
C_3_	Permanent crops	221–223	0.02	0.00	0.00	0.02	0.02	0.00
C_4_	Pastures	231	0.40	0.08	0.39	0.15	0.25	0.15
C_5_	Heterogeneous agricultural areas	241–244	0.08	0.15	0.19	0.11	0.19	0.39
C_6_	Forests and semi-natural areas	311–335	0.15	0.23	0.15	0.23	0.05	0.06
C_7_	Inland wetlands and water bodies	411–412, 511–512	0	0.01	0.02	0.01	0	0.01
C_8_	Marine wetlands and water bodies	421–423, 521–523	0	0	0	0	0	0
	Total		1.00	1.00	1.00	1.00	1.00	1.00

*Source: Coordination de l’Information sur l’Environnment (CORINE) land cover database, 2006 version.; ID, identification code for land-cover class; CLC, CORINE land cover. †Departments: 18, Cher; 41, Loir-et-Cher; 36, Indre; 37, Indre-et-Loire; 49, Maine-et-Loire; 35, Ille-et-Vilaine. ‡Range of CLC classes. Each CLC class is identified by 3 numbers.

### Data Analysis

#### Descriptive Analyses

For each department and species, we computed the herd-level seroprevalence rate (proportion of seropositive herds, i.e., herds with >1 positive serum sample), the animal-level seroprevalence rate, and the distribution of within-herd seroprevalence rates in seropositive herds. For each department and species, we computed a disease reporting rate: the proportion of seropositive herds for which confirmed clinical disease had been reported in 2007 (outbreak status 1). The effects of department, species, and outbreak status on within-herd seroprevalence rate were analyzed with a logistic regression model. A quasi-likelihood estimation procedure was used to account for overdispersion.

#### Seroprevalence in Cattle and Local Conditions in the East–West Transect

We studied the link between local conditions and seroprevalence rate for cattle in the 6 departments of the east–west transect. Seroprevalence data were aggregated at the municipality level. The validity of this aggregation was verified by testing the homogeneity of within-herd seroprevalence rates in municipalities with >1 herd tested. Fisher exact tests were used and Bonferroni correction applied for test interpretation. In case of significant seroprevalence differences between herds, the corresponding municipality was excluded from the dataset. In the remaining municipalities, we computed the municipality-level seroprevalence rate and studied the effects, on this seroprevalence rate, of the following municipality-level covariates: 1) the department, included as a proxy for the municipality relative location in the epizootic wave; 2) the spatial density of cows (number of animals per km^2^), of sheep flocks, and of goat flocks (number of flocks per km^2^); 3) the existence of confirmed clinical cases reported in the municipality in 2007 (binary variable); 4) the proportion of municipality area covered by each land cover class; and 5) the edge density for each pair of land cover classes.

Land cover data were restricted to the 5 classes significantly represented in each of the 6 departments (presence in >50% of the municipalities): C_1_, C_2_, C_4_, C_5_, and C_6_ (Table 3). Variables associated with seroprevalence were identified through a univariate analysis by using logistic models in which the department was systematically included. Variables for which the associated p value was <0.20 were selected for further analysis. A backward elimination process (*8*) was then applied to the corresponding model: variables with the lowest partial F test were successively eliminated until the corresponding p value was <0.05 for each remaining variable. A quasi-likelihood estimation procedure was used throughout. Standardized deviance residuals of the resulting model were tested to detect an association with the department by using the Kruskal-Wallis test. The epidemiologic system studied here is not stationary and clearly isotropic; a significant level of spatial autocorrelation of seroprevalence was thus expected. Since a relatively coarse proxy (the department) was used for representing relative positions in the epizootic wave, regression residuals were likely to be spatially autocorrelated. These residuals were thus examined to determine whether the model captured (or not) the main determinants of local seroprevalence variations. The Geary C statistic was used to quantify spatial autocorrelation of residuals in neighboring municipalities (C = 1: no spatial autocorrelation; C<1: positive autocorrelation; C>1: negative autocorrelation). Significance of this spatial autocorrelation was tested by using a permutation test, with 10,000 random permutations. As a reference, the same test was applied to the residuals of the null model, in which the only independent variable was the department. All statistical analyses were performed by using R software (*9*).

## Results

### Descriptive Results

**Figure 2:**
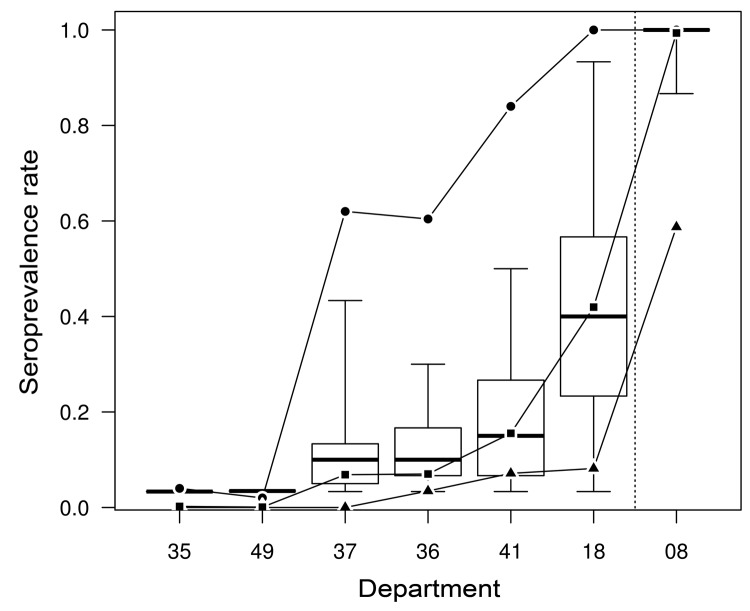
Results from a serologic study of the 2007 epizootic wave of bluetongue virus serotype 8 (BTV-8) in France among cattle herds from an east–west transect of 6 departments (codes 18, 41, 36, 37, 49 and 35) and from the first department to report BTV-8 infection in 2006 (code 08). Circles, herd-level anti–BTV-8 seroprevalence rate; squares, animal-level seroprevalence rate; triangles, proportion of seropositive herds having reported confirmed clinical cases in 2007; box plots, distribution of within-herd seroprevalence rates. Thick horizontal lines represent the median value of the distribution. Rectangles represent the 25th and 75th percentiles. Error bars represent the maximum and minimum values observed in the distribution.

The herds tested in department 08 were seropositive (>1 positive serum test result) except for 2 flocks of sheep (Table 1). Along the east–west transect (Figure 1), the herd-level seroprevalence rate for cattle decreased from 100% in the easternmost department to 4% in the westernmost one (Figure 2). In sheep, herd-level seroprevalence rates were lower than in cattle. For example, in department 18, whereas nearly 100% of cattle herds were seropositive, this proportion fell to 24% in sheep flocks (Table 1). Animal-level seroprevalence rate was close to 100% in cattle of department 08 (Table 2). Along the east–west transect, cattle seroprevalence rate decreased from 42% in the easternmost department to 0.2% in the westernmost one (Figure 2). Seroprevalence rates were lower in sheep: in department 08, nearly 100% of cattle were seropositive, whereas only 57% of sheep were (Table 2). Similar differences were also observed in 2 other departments 18 and, 36. Within seropositive herds, median seroprevalence rate was 100% for cattle herds of department 08, whereas along the east–west transect, it decreased from 40% in the easternmost department to 3% in the westernmost one (Table 2; Figure 2). For each department, this median within-herd seroprevalence rate was close to the animal-level seroprevalence rate (Table 2; Figure 2). Values obtained for sheep flocks were lower.

All herds and flocks included in the study for which confirmed clinical cases had been reported in 2007 were seropositive herds, except for a sheep flock from department 18. In seropositive cattle herds of department 08, the disease reporting rate was 59%. It was much lower along the east–west transect, where it varied from 8% in the easternmost department to 0% in the westernmost departments; however, confidence intervals were wide (Table 1; Figure 2). For a given department, species-specific disease reporting rates were relatively homogeneous: ≈60% in department 08 (59% for cattle and 55% for sheep) and 10% in department 18 (8% for cattle and 14% for sheep).

The logistic model of within-herd seroprevalence rate showed a significant effect of the department (p<0.0001), as expected, because this variable was used as a proxy for the relative location in the epizootic wave. A significant effect of species was also observed with an odds ratio of 0.02 for sheep (p<0.0001, reference: cattle). No significant association between outbreak status and within-herd seroprevalence rate was observed (p = 0.19).

### Seroprevalence in Cattle and Local Conditions in the East–West Transect

The 297 cattle herds of the 6 transect departments were located in 244 municipalities. Most of these contained a single herd, but 42 contained 2 or 3 herds. No significant difference of within-herd seroprevalence was observed in any of these 42 municipalities (Bonferroni correction: p > 0.001), except for 1 municipality in department 41 (p<0.0001). This municipality contained 3 herds, 2 of which had similar seroprevalences (3/30 and 1/30); the third had a higher seroprevalence (15/30). This municipality was excluded from the dataset. Seroprevalence data were then aggregated in the remaining 243 municipalities.

Besides the department, univariate analysis led to selection of the following variables: spatial density of cows, the spatial density of sheep flocks, confirmed clinical cases reported in 2007 in the municipality, proportion of municipality area covered by land cover classes C_4_, C_5_, and C_6_, and edge densities for the following pairs of classes: C_1_–C_4_, C_1_–C_6_, C_2_–C_6_, C_4_–C_5_, C_4_–C_6_, and C_5_–C_6_ (see Table 3 for land cover classes definitions). Following the backward elimination process, only 5 variables were significantly associated with animal-level seroprevalence and kept in the final logistic model (Table 4). The main effect was attributed to the department, with the odds ratio decreasing progressively along the east–west transect. A significant protective effect was associated with the spatial density of cows; however, the strength of this association was moderate (Table 4). The existence of confirmed clinical cases reported in the municipality in 2007 was positively associated with seroprevalence. This positive association was also the case for 2 land cover variables: the edge densities between the forests and seminatural areas (C_6_) class and 2 other classes: pastures (C_4_) and arable land (C_2_) (Table 4). Standardized deviance residuals did not differ significantly between departments (Kruskal-Wallis χ^2^ = 1.96; p = 0.85). No significant aggregation of these residuals was observed (Geary C = 0.58; p = 0.06). Conversely, for the null model (with the department as the only independent variable), the Geary C statistic was lower (indicating a stronger spatial autocorrelation), and a significant aggregation of the standardized deviance residuals was detected (Geary C = 0.46; p = 0.0007).

**Table 4:** Logistic model of animal seropositivity in cattle according to the local conditions in 6 departments aligned on an east–west transect, France, winter 2007–2008*

Variable	Odds ratio	p value
Department		
18: Cher	Reference	
41: Loir-et-Cher	0.21	<0.0001
36: Indre	0.12	<0.0001
37: Indre-et-Loire	0.09	<0.0001
49: Maine-et-Loire	0.002	0.0009
35: Ille-et-Vilaine	0.007	0.0002
Confirmed clinical cases reported in 2007	1.49	0.01
Spatial density of cows in the municipality	0.84†	0.02
Spatial density of sheep flocks in the municipality	NS	0.15
Edge density between arable land and forests or seminatural areas	1.66‡	0.02
Edge density between pastures and forests or seminatural areas	2.06‡	0.001

*NS, not significant. †Change in the odds of seropositivity when spatial density is increased by 10 cows/km^2^. ‡Change in the odds of seropositivity when edge density is increased by 10 m/ha.

## Discussion

Department 08 was the first infected area in France, with the first outbreaks reported there in August 2006. One year later, in winter 2007–2008, this department was behind the epizootic wave, and our results show that, at that time, nearly all cattle were seropositive. Such high seroprevalences have also been observed behind the epizootic wave in Belgium and in the Netherlands (*10**,**11*). Results from the east–west transect show that if the BTV-8 epidemiologic system was clearly saturated behind the epizootic wave, the system was not saturated in or in front of this wave. This transect started from the center of France (in department 18) where, although all the herds housed at least 1 seropositive animal, only 40% of cattle were seropositive (in this department ≈100 outbreaks were reported during October through the end of December 2007). The transect ended in Brittany (in department 35), where 4 of 2,370 cattle were seropositive; each of these 4 animals was located in a different herd (in this department a single outbreak was reported in late November 2007). Similar geographic variations of herd-level and animal-level seroprevalence rates have been observed in Belgium and in the Netherlands (*10**,**11*).

In our survey, seroprevalence variation along the transect showed that within infected areas, herd-level seroprevalence rate increased much earlier than animal-level seroprevalence rate. In cattle in each of the 7 studied departments, the median within-herd seroprevalence rate in seropositive herds was close to the animal-level seroprevalence rates. These results suggest that in cattle the animal infection probability is not particularly higher within infected herds than elsewhere, with seropositive animals being spatially scattered in infected areas rather than clustered inside some herds. The homogeneity of within-herd seroprevalence rates in neighboring cattle herds (located in the same municipality) also supports this hypothesis. Furthermore, if BTV-8 circulation level were more intense and rapid within than between infected herds, seroprevalence rate within seropositive herds should have been homogeneous (and high) along the east–west transect. This was not the case; this seroprevalence rate progressively decreased along the transect.

This absence of within-herd clustering of seropositive cattle could be explained by the fact that these animals spend a large part of the year on pastures. Under favorable temperature conditions, *Culicoides* spp. midges in general (*12*), and species of northern Europe in particular (*13**–**15*), are exophagic (i.e., they feed outside farm buildings) and exophilic (i.e., they rest outside farm buildings). This absence of clustering also suggests that the European epidemiologic system may differ from other systems described in countries where the disease is endemic. In Australia, for example, a close association exists between *C. brevitarsis* midges and cattle in whose dung *Culicoides* spp. midges lay eggs and larvae develop (*16**,**17*). Such a close association does not seem to exist in Europe, where contradictory results have been recently published about the role of cattle dung in the life cycle of local *Culidoides* spp. midges (*18**,**19*). Seroprevalence rates in sheep were similar to those obtained in the Netherlands during winter 2006–2007 (*20*). Sheep were globally less frequently infected than cattle. In department 08, the only fully seronegative herds were 2 sheep flocks, and logistic modeling attributed a strong protective effect to this species. These results reflect the trophic preferences of vectors (*21*).

Except in department 08, no clinical cases were reported in most seropositive herds or flocks in 2007. The proportion of these silently infected herds was 40% in department 08, and >90% in the east–west transect. At the herd level, no association was detected between occurrence of clinical cases and seroprevalence rate. However, this association was observed in cattle at the municipality level. In the east–west transect, confirmed clinical cases locally reported in 2007 (whatever the species of diseased animals) increased the animal-level seroprevalence risk. Clinical cases caused by a higher (and possibly longer) viremia could increase local viral circulation. This interpretation does not contradict the absence of association at the herd level. If clinical cases increase BTV-8 circulation not only inside the affected herd but also in the neighboring ones, association between seroprevalence rate and occurrence of clinical cases is then much more difficult to demonstrate at the herd level. A protective effect of the local density of cows was observed. The low infection rate of *Culicoides* spp. under experimental (*22*) or field conditions (*23*) could explain this result: increased cattle densities would dilute infective bites and decrease individual infection risk, with seroprevalence being consequently lower.

Two land cover variables were identified as seropositivity risk factors; both are edge densities. These landscape indices are increased when the interweaving of land cover themes increases because landscape is complex and fragmented. Fragmentation indices have been linked to abundance of vectors of BTV (*C. imicola* midges in southern France) (*24*), and the local density of forest and pasture edges have been linked to bluetongue disease risk in Corsica (*25*). More generally, ecotones are places where vegetal and animal species of different ecosystems meet and mix and thus where contact rates are increased. Therefore, landscape indices are considered as useful tools for biodiversity assessment (*26*), and species richness of several animal groups (in particular arthropod groups) has been linked to landscape heterogeneity (*27*). In our study, edges between pastures and forests could be areas where species richness is greater, both for vectors (*Culicoides* spp. midges) and for hosts (wild and domestic ruminants). This species richness could allow a more intense BTV circulation than elsewhere, inducing a higher seroprevalence in cattle.

The positive association between seroprevalence in cattle and the edge density between forests and arable land is more difficult to interpret. These edges could represent borders between resting (forest) and feeding (arable land) areas for wild ruminants. An increased density of these edges could then indicate an increased carrying capacity of wild ruminant species, in turn supporting an increased local BTV circulation. Several recent studies have demonstrated high anti-BTV seroprevalence levels in wild ruminants. In BTV-4–infected areas in Spain, a seroprevalence of 66% was observed in red deer (*Cervus elaphus*) (*28*). Interestingly, in this species, seroprevalence rate was higher in free-ranging animals than in captive ones (*29*). In Belgium, a seroprevalence of 52% was observed in red deer in 2007 (*30*). In France, a similar seroprevalence (41%) was observed in this species during winter 2008–2009; seroprevalence reached 70% in some areas (*31*). These field studies and the results of our study suggest that BTV circulation in Europe could involve complex epidemiologic cycles with several host and vector species. As suggested elsewhere (*28*), thorough research is needed on hosts and vectors involved in BTV circulation in natural ecosystems in Europe.
